# Understanding the physiological roles of the neuronal calcium sensor proteins

**DOI:** 10.1186/1756-6606-5-2

**Published:** 2012-01-23

**Authors:** Robert D Burgoyne, Lee P Haynes

**Affiliations:** 1 Department of Cellular and Molecular Physiology, The Physiological Laboratory, Institute of Translational Medicine, University of Liverpool, Liverpool, L69 3BX, UK

**Keywords:** Calcium, Calcium-sensor, EF-hand, neuronal signalling, protein complex, protein structure

## Abstract

Calcium signalling plays a crucial role in the control of neuronal function and plasticity. Changes in neuronal Ca^2+ ^concentration are detected by Ca^2+^-binding proteins that can interact with and regulate target proteins to modify their function. Members of the neuronal calcium sensor (NCS) protein family have multiple non-redundant roles in the nervous system. Here we review recent advances in the understanding of the physiological roles of the NCS proteins and the molecular basis for their specificity.

## Introduction

Calcium has a major role in neurons as the trigger for neurotransmitter release [1,2]. In addition many other aspects of neuronal function are regulated by changes in intracellular free Ca^2+ ^concentration. Rapid exocytosis of neurotransmitter-containing synaptic vesicles is activated by a local increase in Ca^2+ ^concentration at the presynaptic active zone within 10 s of microseconds [3] through the action of the Ca^2+ ^sensor synaptotagmin [4]. Other Ca^2+^-regulated events require more global changes in neuronal Ca^2+ ^concentration, are activated over longer time scales and the changes can persist for minutes to days [5]. In part the specificity of the effects of Ca^2+ ^on neuronal physiology is determined by the magnitude, kinetics and spatial localisation of the Ca^2+ ^signal [6]. The transduction of changes in Ca^2+ ^concentration requires Ca^2+^-binding proteins and these can contribute to the overall specificity of Ca^2+ ^signalling. One well-characterised Ca^2+^-binding protein with neuronal functions is the ubiquitous protein calmodulin which binds Ca^2+ ^through its four EF-hand domains [7]. Other EF-hand containing proteins have been found to be expressed in neurons and these contribute to the diversity of the neuronal Ca^2+ ^signalling repertoire. These include two families known as the CaBPs/calneurons [8-13] and the neuronal calcium sensor (NCS) family. The latter family are the subject of this review. The NCS proteins have a wide range of physiological functions in neurons and in the photoreceptor cells in the retina. An emerging area is the recognition of the importance of NCS protein signalling in neuronal disease [14-20].

A key issue regarding the NCS proteins is how they can differentially affect specific aspects of neuronal function. NCS protein function is established by several factors determined by their intrinsic properties including their ability to interact with and regulate different target proteins. Several reviews on the NCS proteins have been published [21-25] and here we will concentrate on recent advances in the understanding of their physiological roles and the underlying target protein interactions that determine their specific functions.

## Overview of the NCS protein family

The NCS proteins are encoded by 14 conserved genes in mammals and their diversity is increased by the existence of splice variants that are likely to modify their functions in ways yet to be fully determined [21,23]. The NCS family consists of NCS-1, hippocalcin, neurocalcin-δ, VILIP1-3, recoverin, GCAP1-3 and KChIP1-4. The core EF-hand containing regions of these proteins are similar but they are largely distinguished by variable N-terminal and C-terminal domains. Genetic studies in various organisms have shown that loss of one of the NCS proteins can result in a distinct, detectable phenotype suggesting that they do not overlap in function sufficiently for the loss to be compensated by another family member or by calmodulin which can regulate some of the same targets [23]. Recoverin and the GCAPs have well-defined roles in the regulation of phototransduction and are only expressed in the retina [26,27]. The lack of redundancy of the other NCS proteins could come from restricted expression in particular classes of neurons but this is likely to be only a part of the explanation as many neurons appear to express multiple NCS proteins. Other factors that contribute to their non-redundancy include a higher Ca^2+ ^sensitivity than calmodulin suggesting that they respond to different Ca^2+ ^signals. They have varied subcellular locations through the post-translational modifications of myristoylation [28,29] or palmitoylation [30] that allow constitutive or alternatively Ca^2+^-dependent membrane association. In addition, they have specific binding targets for regulation.

## Physiological functions of the NCS proteins

### Recoverin

As indicated above, recoverin has a characterised function in the retina [31] and indeed is only known to be expressed in photoreceptors, retinal cone bipolar cells and the pineal gland [32]. Recoverin's physiological role was suggested to be to regulate light sensitivity [31,33]. It does this through the direct inhibition of the retinal rhodopsin kinase in a Ca^2+^-dependent manner [32,34,35]. The normal phosphorylation of rhodopsin is crucial for the switch-off of the light response in photoreceptors [36]. A physiological role for recoverin in the regulation of light sensitivity has been established through study of mice where recoverin expression is reduced or knocked-out [31,33]. Rhodopsin kinase is recoverin's main known target [35,37] although it has also been found exert an additional, less characterized, effect on light sensitivity apparently independently of the kinase [38]. In addition it has been shown to be able to recruit the Ca^2+^-binding protein caldendrin to membranes [39]. Recoverin was the first of the NCS proteins to be discovered [40] and has since been extensively characterised from a biochemical perspective. The precise molecular basis for the inhibition of rhodopsin kinase [37] has been established through structural analyses (see below).

### GCAPs

The GCAPs [27,41] have role in light adaptation [42,43] by activating or inhibiting retinal guanylyl cyclases [44,45] at low or high Ca^2+ ^concentration respectively [46]. The importance of the GCAPs is illustrated by the fact that various mutations in GCAP1 in humans result in cone dystrophies with photoreceptor degeneration [47]. The physiological importance of the GCAPs in the retina has also been established through knock-out of GCAP1 and GCAP2 in mice [42,48-50]. One key issue is why multiple GCAPs are required for light adaption. For example, GCAPs 1 and 2 are expressed in the same photoreceptor cells. Expression of GCAP2 in mice lacking GCAP1 and 2 did not result in full recovery of function indicating non-redundancy of function [50]. The existence of multiple GCAPs appears to be related to a need for sensors with differing Ca^2+ ^sensitivities to allow responses to occur over a wide range of Ca^2+ ^concentrations [27,51]. The structures of GCAP1 1, 2 and 3 have been characterised [52-55] but the molecular details of how they regulate guanylyl cyclases are yet to be determined.

### NCS-1

**Figure 1:**
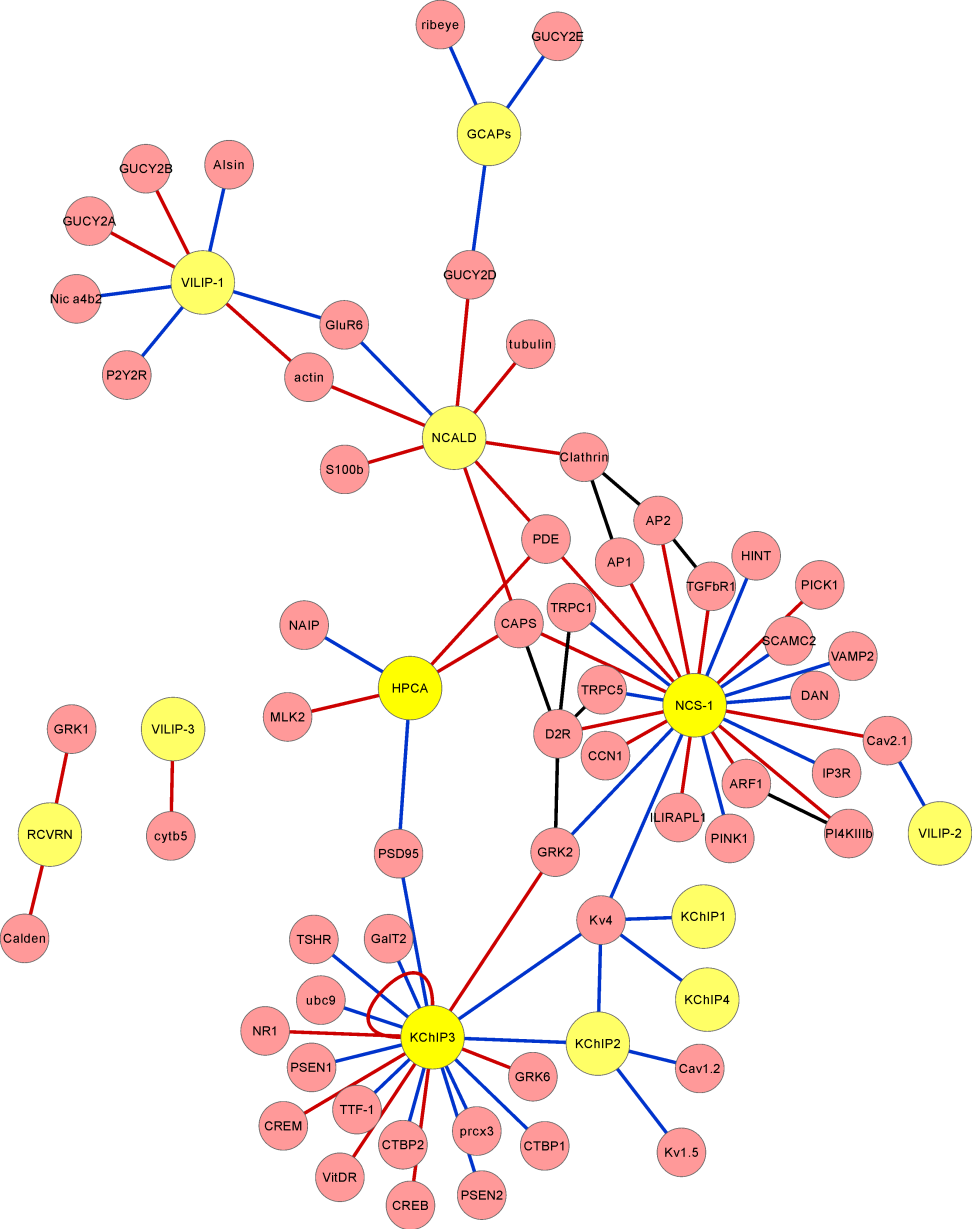
**An interaction map of the known binding partners of the NCS proteins**. The NCS protein family members are shown in yellow and binding partners in red with interactions directly with NCS proteins or between binding partners indicated. The edge colours indicate whether the interaction are with the Ca^2+ ^-bound form (red) or the apo form (blue) or if this is not applicable (black). The binding partners and their abbreviations are as follows: Actin, Alsin, AP1 (clathrin adaptor), AP2 (clathrin adaptor), ARF1 (ADP-ribosylation factor-1), Calden (Caldendrin), CAPS (Ca^2+^-dependent activator protein for secretion), Cav1.2 (Ca^2+ ^channel), Cav2.1 (Ca^2+ ^channel), CALN (Calcineurin), Clathrin (Clathrin heavy chain), CREB (cAMP responsive element binding protein), CREM, (cAMP response element modulator), CTBP1/2 (C-terminal binding protein 1), cytb5 (cytochrome b5), D2R (dopamine D2 receptor), DAN (differential screening-selective gene aberrant in neuroblastome), GalT2 (galactosyl transferase 2), GluR6 (glutamate receptor 6), GRK1/2/6 (G protein-dependent receptor kinase 1/2/6), GUCY2A/2B/2D/2E (guanylate cyclase 2A/2B/2D/2E), HINT (histidine triad protein), ILIRAPL1 (interleukin receptor accessory protein like-1), IP3R (inositol 1, 4, 5 trisphosphate receptor), Kv1.5 (potassium channel), Kv4 (potassium channel), MLK2 (Mixed lineage kinase 2), NAIP (Neuronal apoptosis inhibitory protein), Nic a4b2 (nicotinic receptor containing 4 α and 2 β subunits), NR1 (NMDA receptor type 1), P2Y2R (purinergic receptor type 2Y2R), PDE (phosphodiesterase 1A), PI4KIIIb (phosphatidylinositol-4-kinase IIIβ), PICK1 (Protein interacting with C kinase 1), PINK1 (PTEN-induced kinase), prdx3 (Peroxiredoxin 3), PSD95 (post-synaptic density protein 95), PSEN1/2 (presenilin 1/2), ribeye (synaptic ribbon protein), S100b (S100 protein beta), SCAMC2 (Short calcium binding mitochondrial carrier 2), TGFbR1 (transforming growth factor β receptor type 1), TRPC1/5 (transient receptor potential channel 1/5), TSHR (thyroid stimulating hormone receptor), TTF-1 (Thyroid transcription factor 1), tubulin (α and β tubulin), ubc9 (Ubiquitin conjugating enzyme 9), VAMP2 (vesicle -associated membrane protein 2), VitDR (vitamin D receptor). Note that for simplicity we have used the name KChIP3 despite the fact that several of the original descriptions of the interactions used the alternative terminology of DREAM or calsenilin (see main text).

The other NCS proteins have been implicated in a wide range of physiological functions in neuronal cells [23]. NCS-1 has been studied in various different organisms as it is expressed from yeast to man. This has resulted in it being implicated in many functional roles ranging from regulation of neuronal ion channels, membrane traffic, learning, neuronal growth and survival [23,25]. These functions appear to involve interaction of NCS-1 with multiple target proteins [17,56-59] many of which are apparently specific for this member of the family (Figure 1). Recent physiological studies have characterised several new roles for NCS-1 working through different pathways. Earlier work had suggested a role for NCS-1 in regulation of neurotransmitter release [60-62], voltage-gated Ca^2+ ^channels [63,64] and in short-term synaptic plasticity [65]. More recently NCS-1 has been shown to act in a pathway, involving interactions with another Ca^2+ ^sensor PICK1, that mediates long-term depression (LTD) in rat cortical neurons [66]. In the mouse, NCS-1 has been implicated in exploratory behaviour and in the acquisition of spatial memory by regulating the surface expression of dopamine D2 receptors in the hippocampal dentate gyrus [67]. A study in *Drosophila *has shown that the fly NCS-1 orthologue frequenin which regulates both neurotransmitter release [60] and nerve terminal growth [68] does so through an interaction (revealed genetically) with the voltage-gated Ca^2+ ^channel *cacophony *[69]. This channel is the fly equivalent of the mammalian P/Q-like Cav2.1 channel which is also regulated by calmodulin [70] and the other Ca^2+^-binding proteins VILIP-2 [71] and CaBP1 [72]. Overexpression of NCS-1 was demonstrated to induce neurite sprouting and spinal cord regeneration [73]. This appeared to involve activation of the PI3K/Akt pathway but it is not known whether NCS-1 directly interacts with proteins of this pathway. Finally, recent work has suggested that NCS-1 has a positive role in increasing Ca^2+ ^signalling in cardiac cells through its interaction with the IP3 receptor [74,75]. It is not clear, however, whether NCS-1 has a physiological role in regulating IP3 receptors in neurons.

### Hippocalcin and VILIPs

Hippocalcin is expressed at highest levels in hippocampal neurons [76,77] and hippocalcin-deficient mice show an impairment in memory formation [78]. Hippocalcin has been suggested to be involved in LTD in hippocampal neurons by regulating endocytosis of the GluR2 class of AMPA glutamate receptors based on a direct interaction with the endocytotic clathrin adaptor AP2 [79]. More recently it has been shown to act via AP2 and the post-synaptic protein PSD-95 in a pathway for LTD triggered by muscarinic receptor activation [80]. Hippocalcin has also been suggested to regulate neuronal function as the calcium sensor for the potassium channels that lead to hyperpolarisation known as the slow after-hyperpolarisation currents (sAHP) [81,82]. The closely related NCS protein neurocalcin δ also regulates the sAHP [82]. Hippocalcin has a myristoyl switch that allows its reversible membrane association on Ca^2+ ^binding [83]. This switch can be activated by Ca^2+ ^influx through synaptic NMDA receptors to result in rapid translocation of hippocalcin into dendritic spines [84]. This translocation of hippocalcin may be important for LTD or the activation of sAHP. The VILIPs that are also closely related to hippocalcin may have other physiological roles [24] through regulation of voltage-gated Ca^2+ ^channels [71], nicotinic acetylcholine receptors [85,86] or purinergic P2X2 receptors [87]. Recent structural analyses suggest that VILIP-1 may function as a dimer [88,89]. VILIP-2 regulates the function of Cav2.1 calcium channels that are also regulated by calmodulin. Calmodulin binding to the α-subunit of these channels is required for both Ca^2+^-dependent facilitation and inactivation of the channels [90,91]. In contrast, VILIP-2 increases facilitation but slows inactivation of the channels [71]. Recent work [92] has used calmodulin/VILIP-2 chimeric proteins and has determined which domains in VILIP-2 are responsible for the differing effects of VILIP-2 compared to calmodulin. Distinct contributions of the N and C-terminal regions of VILIP-2 to the regulation of the channels were identified.

### KChIPs/DREAM/calsenilin

The KChIP proteins were so named based on their ability to interact with the Kv4 family of A-type rapidly-inactivating potassium channels [93]. One of the KChIIPs, KChIP3 was originally discovered in 1998 as a protein that bound to the presenilin proteins and was named calsenilin [94]. The characterisation of a protein that affects gene transcription through binding to a specific regulatory DNA motif (the downstream regulatory element or DRE) led to the identification of a human protein that was named downstream regulatory element antagonist modulator (DREAM) [95]. In the initial publication this protein appeared to have an alternative start codon giving a novel 30 residue extension at the N-terminus. This sequence was subsequently corrected in the databases and it is clear that calsenilin, DREAM and KChIP3 proteins are in fact identical. The continued use of all three names is potentially confusing and so here we will refer to the protein as KChIP3. This is for simplicity and also since KChIP3 is closely related to KChIPs 1, 2 and 4 forming the KChIP sub-family of the NCS proteins.

In addition to the four KChIPs originally identified that are coded by 4 distinct genes there are multiple splice isoforms expressed [96]. The majority of the KChIP isoforms that have been examined regulate the gating properties of Kv4 channels [93] and also stimulate their traffic through the secretory pathway to the cell surface [93,97-99]. In contrast, certain isoforms of KChIPs 2, 3 and 4 inhibit Kv4 channel traffic to the plasma membrane due to the existence of an alternative N-terminal inhibitory domain (the KIS domain) [100]. This hydrophobic sequence has been suggested to be a trans-membrane domain [101] but structural characterisation has shown that the N-terminal helix of KChIP4a instead binds within the hydrophobic pocket of KChIP4a to displace the N-terminus of Kv4 [102,103] explaining its dominant inhibitory effect on Kv4 channel traffic.

Recent work has suggested that KChIPs may also play a role in the regulation of voltage -gated Ca^2+ ^channel signalling. T-type (Cav3) Ca^2+ ^channels were found to be present in a signalling complex containing Kv4 channels and Ca^2+ ^entry though the Cav3 channels modified the gating of the Kv4 potassium channels. This effect was mediated by KChIP3 as the Ca^2+ ^sensor and interestingly this could not be replaced by KChIPs 1, 2 or 4 providing further evidence for specific actions of the different KChIPs [104]. In addition, to this study, it has also been recently suggested that KChIP2 is a directly interacting regulator of L-type (Cav1, 2) Ca^2+ ^channels and that KChIP2 can increase channel density without an effect on channel traffic [105].

The multiple possible roles of KChIP3/calsenilin/DREAM that gave rise to the independent identifications have been supported by studies in KChIP3 knock-out mice [106,107] It is clear that KChIP3 regulates expression of multiple genes in addition to its other cellular roles [108]. The gene regulatory function (DREAM) involves in part its direct interaction with the DNA DRE motif but growing evidence has shown its interaction with several co-repressor proteins involved in gene regulation [109-111].

The interaction of KChIP3 (calsenilin) with the presenilins [94] has been of interest given the importance of mutations in the presenilins in familial forms of Alzheimer's disease [112]. The full significance and the physiological relevance of the interaction of KChIP3 with the presenilins remains to be established, however, although both earlier [113] and more recent work has suggested an important role in Ca^2+ ^signalling [114]. In addition, it has been suggested that the interaction of KChIP3/calsenilin could regulate the processing function of presenilin/γ-secretase [115,116].

Further analysis of transgenic mice has identified additional physiological neuronal roles for KChIP3. From two independent studies on the knock-out mouse there is evidence for an enhancement of learning (contextual fear memory) in the absence of KChIP3 [117,118]. One study found an increase in long-term potentiation (LTP) in the hippocampus of the knock-out mice [117]. In a study of mice expressing a Ca^2+^-insensitive active mutant form of DREAM learning was impaired consistent with the knock-out mouse studies. In contrast to the knock-out mouse data however no effect on LTP was observed but instead LTD was reduced [119]. The effect of DREAM on LTD was suggested to be due to an interaction with the post-synaptic protein PSD-95 that could affect NMDA receptor function [119]. Another study has identified a direct interaction of KChIP3 with the NR1 subunit of the NMDA receptor [120]. This interaction inhibits the surface expression of NMDA receptors and could contribute to its inhibitory effect on learning and memory. Another important role for KChIP3 in neuronal physiology has come from other studies of the mice expressing the Ca^2+^-insensitive constitutively active mutant. This work has identified a requirement for KChIP3 in central nervous system mechanisms of pain sensation and this involved the action of BDNF (brain-derived neurotrophic factor) [121].

A role for KChIP1 in synaptic plasticity and behaviour has also been revealed from the study of a mouse KChIP1 knock-out. KChIP1 was found to be expressed at high levels in GABAergic interneurons [122]. In the absence of KChIP1, abnormalities in GABAergic neurotransmission and an increase in anxiety-related behaviours was identified [123]. In contrast to the positive role of KChIP1 in stimulating traffic of Kv4 channels, the KChIP1 knock-out study found an increase in potassium channel density which could be related to an inhibitory effect of KChIPs that has been observed for traffic of Kv1.5 potassium channels to the plasma membrane [124].

Apart from the distinct effects of some KChIP isoforms on Kv4 channel traffic noted above there is relatively little information on why there are so many of them (potentially at least 16 isoforms [96]). One study detected a specific effect of over-expressed KChIP3 compared to single isoforms of KChIP1, 2 and 4 on Ca^2+ ^signalling and neurotransmitter exocytosis [98]. There is evidence that different classes of neurons express different subsets of KChIP proteins [125,126]. One recent study showed that KChIPs 2, 3 and 4 can all contribute to Kv4 channel function in the same mouse cortical pyramidal neurons [127]. In contrast, another study found that Kv4 potassium currents in hippocampal interneurons were largely dependent on only KChIP1 [128]. Another possible clue explaining the reason for multiple KChIPs may the existence of differing neuronal signalling complexes involving Kv4 channels and KChIPs [104,129]. It is unclear whether the KChIP isoforms have specific functions due to interaction with distinct target proteins. An increasingly large number of targets are being identified for KChIP3 [130] but less is known about the targets of the other KChIPs (Figure 1).

## Structural insights into NCS protein function and target specificity

The structures of a number of NCS protein family members have been solved by the use of X-ray crystallography or NMR spectroscopy. These variously include structures of apo and Ca^2+ ^-bound forms with or without N-terminal myristoylation [131]. While the NCS proteins have similar overall molecular structures there are differences that provide clues to their specificity of function and more specifically their target protein interactions. They all possess four EF-hand domains but in each case EF hand 1 is inactive in binding divalent cations. The Ca^2+^-bound forms of the NCS proteins either have 3 bound Ca^2+ ^ions or in the case of recoverin [132], KChIP1 [133,134] and KChIP3 [135] have 2 bound Ca^2+ ^ions and these proteins differ in which EF hands are active. Many but not all of the NCS proteins are N-terminally myristoylated to allow membrane association and the use of the myristoylation confers different properties [136,131]. The structurally most extensively characterised NCS protein is recoverin. This protein has a well defined Ca^2+^/myristoyl switch mechanism in which the myristoyl group is sequestered in the Ca^2+ ^free state and is flipped out on Ca^2+ ^binding to allow membrane association [132,137]. A dynamic Ca^2+^/myristoyl switch mechanism allowing reversible membrane association has been demonstrated in live cell studies for hippocalcin [28,83,84] and the VILIPs [138,139]. In contrast, NCS-1 appears to have a solvent exposed myristoyl group that drives its constitutive membrane association [28,29] although it does dynamically cycle between the membrane and cytosol [18]. A further important aspect of the myristoyl group is that it is not sequestered identically in all characterised structures [53,132,140] and can affect the overall protein structure and stability [55,141]. An additional aspect of the NCS proteins that may be crucial for the specificity of target interactions is that they have a very variable distribution of charged residues particularly on their C-terminal halves [131].

**Figure 2:**
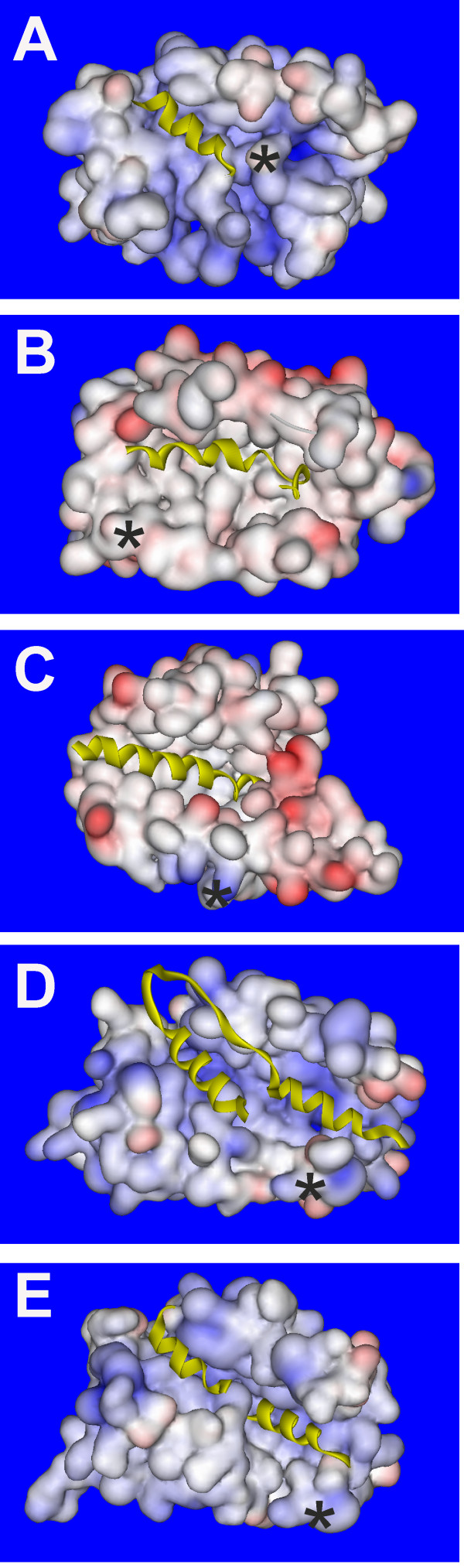
**Structures of NCS proteins showing interactions within the exposed hydrophobic groove**. (A) Structure of Ca^2+^-bound bovine recoverin with bound N-terminal fragment of rhodopsin kinase including residues 1-25 ([37]; PDB, 2I94). Recoverin is shown in space-filling representation and the rhodopsin kinase is shown in yellow. The rhodopsin kinase fragment forms an α-helix that is embedded in an N-terminal hydrophobic pocket of recoverin. The asterisk indicates the position of the C-terminus of recoverin. (B) Structure of Ca^2+^-bound human KChIP1 showing the binding of the N-terminal residues 3-27 of Kv4.3 ([143,144]; PDB, 2I2N). KChIP1 is shown in space-filling representation and the Kv4.3 fragment which forms an α-helix shown in yellow is embedded in a hydrophobic groove running across KChIP1. An additional interaction site of Kv4.3 with helix H2 of KChIP1 is omitted for clarity. The asterisk indicates the position of the C-terminus of KChIP1. (C) Structure of of Ca^2+^-bound mouse KChIP4a ([102];PDB, 3DD4) with the structure shown in space-filling representation apart from the N-terminal residues 1-23 which are shown in yellow. The N-terminus forms an α-helix which is embedded within the N-terminal part of the exposed hydrophobic groove of KChIP4a. The asterisk indicates the position of the C-terminus of KChIP4a. (D) Structure of of Ca^2+^-bound *S. cerevisiae *Frq1 with bound Pik1(121-175) ([142];PDB, 2JU0) the structure of Frq1 is shown in space-filling representation and the Pik1 fragment in yellow. Pik1 forms two α-helices joined by a loop and the helices are bound to the N- and C-terminal parts of a large hydrophobic groove running across Frq1. The asterisk indicates the position of the C-terminus of Frq1. (E) Model for the structure of of Ca^2+^-bound human NCS-1 with two bound molecules of the C-terminal peptide of the D2/D3 receptor. The structure of NCS-1 derived from PDB 1G8I[149] is shown in space-filling representation and the D2/D3 peptides in yellow. The two peptides are bound to the N- and C-terminal parts of the large hydrophobic groove running across Frq1. The asterisk indicates the position of the C-terminus of NCS-1.

Insight into the molecular basis of NCS protein specificity has come from structural characterisation of NCS/target complexes (Figure 2). The structures have been solved for the complex of recoverin with a rhodopsin kinase fragment [37], the frequenin/Pik1 complex from *Saccharomyces cerevisiae *and *Schizosaccharomyces pombe *[140,142] and a complex of KChIP1 with the N-terminus of the Kv4.3 channel [143,144]. Additional information has also come from the structure of KChIP4a, the role of which is to inhibit fast inactivation of the Kv4 channel and to reduce its traffic to the plasma membrane through its specific K^+ ^channel inactivation suppressor (KIS) domain [100,97]. This structure showed [102] that the N-terminal KIS domain is sequestered in the hydrophobic groove that in KChIP1 is involved in interaction with the Kv4 channel and can thereby compete off this interaction [143,144]. The consequence is that unlike KChIP1, KChIP4a would only be able to interact with Kv4 through the additional site 2 interaction identified for KChIP1/Kv4.3 [143,144]. In addition, a model has been generated for the interaction of NCS-1 with dopamine D2/D3 receptors [145]. NCS-1 regulates the internalisation of D2 and D3 receptors through interaction with the short cytoplasmic C-terminus of the receptor [59]. A synthetic peptide based on this sequence directly binds to NCS-1 [145,146] allowing characterisation of the interaction by use of NMR. The key features that have emerged from these studies are the importance of an exposed hydrophobic groove lined by conserved hydrophobic residues for binding α-helical regions of the target protein, the importance of non-conserved residues in specific target contacts and the differential role of the NCS protein C-terminus in target interactions.

All of the characterised interactions shown in Figure 2 involve α-helix binding within a hydrophobic groove in the N-terminal half of the NCS protein. In the case of frequenin [140,142] and predicted for NCS-1 [145], two α-helices are able to bind to separate hydrophobic pockets in a much longer exposed cleft. The exposure of the C-terminal part of the groove would require significant movement of the C-terminal α-helix of the NCS protein [142,145]. In a recent paper is has been proposed that the C-terminus of NCS-1 binds directly to the hydrophobic groove as a mimic of the target ligand to stabilise the structure of NCS-1 [147]. A movement of the C-terminal H10 helix of KChIP1 was observed in the KV4.3 bound structure [143,144] compared to the unbound form of KChIP1 [134] that would allow the single α-helix of Kv4.3 to interact with hydrophobic residues more C-terminal than is the case for the recoverin/rhodopsin kinase complex. (Note that Kv4.3 also makes a second contact with the H2 helix of KChIP1 that is not shown in Figure 2[143,144]). In the recoverin/rhodopsin kinase complex the C-terminal helix of recoverin occludes the hydrophobic cleft and indeed residues in the C-terminus are crucial for the interaction with the target [148]. From the structures available it is possible to see how a similar mechanism for target interaction through binding of a target α-helix into an exposed hydrophobic cleft has been modified in each protein to generate high specificity for target binding.

## Conclusions and Future Directions

There is an increasing understanding of the importance and diverse functional roles of the NCS proteins in transducing neuronal Ca^2+ ^signals. It is clear that additional roles will continue to emerge. Additional analysis of the protein targets for the NCS proteins and the structural characterisation of these interactions will allow us to further understand the molecular basis for the specificity of the NCS protein function. In addition, further study will be required to fully define the importance of NCS protein signalling, or disorders in this signalling, in neuronal disease.

## Competing interests

The authors declare that they have no competing interests.

## Authors' contributions

RDB drafted the manuscript. RDB and LPH revised the manuscript. RDB and LPH read and approved the final manuscript.
